# Combined Effect of Cultivar and Peel Chromaticity on Figs’ Primary and Secondary Metabolites: Preliminary Study Using Biochemical and FTIR Fingerprinting Coupled to Chemometrics

**DOI:** 10.3390/biology10070573

**Published:** 2021-06-23

**Authors:** Lahcen Hssaini, Kaoutar Elfazazi, Rachid Razouk, Rachida Ouaabou, Francisca Hernandez, Hafida Hanine, Jamal Charafi, Karim Houmanat, Rachid Aboutayeb

**Affiliations:** 1 National Institute for Agricultural Research (INRA), P.O. 415, Rabat 10000, Morocco; ka.elfazazi@gmail.com (K.E.); jcharafi@gmail.com (J.C.); k.houmanat@gmail.com (K.H.); rachid.engineer@gmail.com (R.A.); 2 Faculty of Sciences Semlalia, Cadi Ayyad University, P.O. 2390, Marrakesh 40000, Morocco; rachaouaabou@gmail.com; 3 Grupo de Investigación en Fruticultura y Técnicas de Producción, Centro de Investigación e Innovación Agroalimentaria y Agroambiental (CIAGRO-UMH), Miguel Hernández University, Carretera de Beniel, km 3.2, 03312 Orihuela, Spain; francisca.hernandez@umh.es; 4 Laboratory of Bioprocessing and Bio-Interfaces, Faculty of Sciences and Technics, Université Sultan Moulay Slimane, P.O. 523, Beni-Mellal 23000, Morocco; hafidahanine0@gmail.com

**Keywords:** secondary metabolites, antioxidant activity, FTIR fingerprinting, chemometrics, *Ficus carica* L.

## Abstract

**Simple Summary:**

Primary and secondary metabolites are among the markers for addressing fig chemotypic variability. These compounds are mainly driven by the cultivar factor besides chromatic coordinates color as they are highly correlated to these biomarkers. Combined use of chemical analytical procedures and vibrational spectroscopy is of great importance for a better understanding of network connections within the dataset. In this study, we screened 11 fig tree cultivars for their biochemical and spectral fingerprints in a comparative scheme for high resolution discrimination. Analytical methods herein used were also evaluated for their greenness aspects using GAPI eco-scale tool.

**Abstract:**

Figs are a traditional pantry staple for healthy eating in Middle Eastern and North African countries as fig trees grow abundantly in such hot and dry climates. Despite the importance of this species, chemotypic diversity has gone unheeded and therefore its valorization pathways remain poorly documented. For this reason, high-pressure liquid chromatography (HPLC) alongside vibrational spectroscopy were used to investigate the changes of antiradical potency and primary and secondary metabolites in fresh figs with regard to the combined effect of the cultivar factor and the fruit peel chromatic coordinates. Fourier-transform infrared spectroscopy (FTIR) fingerprinting displayed six major peaks assigned to functional groups of the investigated samples with significant differences in their vibration intensities. Biochemical screening revealed highly significant variability (*p* < 0.05) among the investigated cultivars. Antioxidant activity was found to be higher in free radical scavenging using 2,2-diphenyl-1-picrylhydrazyl (DPPH) compared to ferric reducing ability (FRAP). Chemometric investigations of both biochemical and FTIR fingerprinting showed satisfactory resolutions, and the total phenol contents and chromatic coordinates had the highest scores in the dataset. However, the cultivars’ geographical origin seemed not to have a clear impact on the clustering results. The aforementioned analytical procedures were found to be equally important and can be jointly used for high-resolution screening and discrimination of fig trees.

## 1. Introduction

In Morocco, a country with rich fig (*Ficus carica* L.) biodiversity and the third largest fig producer worldwide, this species’ valorization remains poorly controlled and less documented [1]. Despite being an important and highly nutritive commodity, figs are so far one of the less studied species in Morocco. Their chemotypic diversity has largely gone unheeded for many reasons, some of which have to do with their wild aspect, since they still naturally growing in mountains and family gardens, and subsequently remain poorly exploited [2,3]. Moreover, figs are easily perishable fruits, for which preservation of quality and health-promoting components is most often costly, as it requires sophisticated chain distribution and storage facilities [4]. To date, there are very few studies on figs’ potential and chemotypic diversity in Morocco, and thus selection for industrial valorization remains less developed [5,6]. 

Figs and their special type of inflorescence, called *syconium*, are a typical food of Mediterranean populations [7]. They are an important source of bioactive compounds [8]. Their consumption is mainly due to their taste, flavor and health-promoting properties, which are related essentially to their high levels of sugars, trace minerals, fibers, vitamins and antioxidant potency, resulting mainly from their phenolic profile [8,9]. Figs are known to have a high commercial value, due particularly to their excellent taste, flavor and nutritional attributes. They are also appreciated for their taste and sweetness, which can be influenced by their sugar and organic acid compositions [9]. Recently, interest in fig consumption has significantly increased due to their health-supporting metabolites [5,6,7,8,9,10]. The latter are in particular related to the high amounts of anthocyanins, flavonols and other polyphenolics that contribute to their high antioxidant activity [1]. Polyphenolic compounds are generally higher in fig cultivars with dark-colored peel, which contain higher levels of antioxidants compared to fig accessions with light-colored peel [5,6,7,8,9,10,11]. Figs are rich in sugars and organic acids [5]. Indeed, several studies reported that glucose and fructose are the main sugars in fig fruits [12,13,14,15]. Only minor concentrations or even traces of sucrose have been measured in analyzed fig fruit [9,10,11,12,13,14,15]. According to some reports, sucrose was not determined in some accessions. Indeed, sugars composition and amounts may influence fruit sweetness and taste [1]. Malic and citric acid were identified as the major organic acids in figs. However, fumaric, oxalic, succinic and shikimic acids were also reported in minor amounts [9,14,16]. The accumulation of primary and secondary metabolites in fig trees is strongly dependent on environmental conditions and is also genetically controlled [6,9,17].

The aforementioned constituents are usually assessed using complex technologies such as method-based chromatography separation, which involves high-cost wet chemistry techniques and requires laborious work and time. Therefore, particular attention has been given to vibrational spectroscopy techniques, which are highly accurate in providing different levels of molecular information regarding primary and secondary metabolite structures [18].

With the rise of high-throughput assay technologies, both biochemical and vibrational spectroscopy fingerprinting approaches have every so often been coupled with chemometric techniques to determine relevant biomarkers and better understand the multiple associations between these attributes [19]. Clustered heatmaps along with principal component analysis (PCA) are thus among the widespread methods for assessing biochemical data. In this study, high-pressure liquid chromatography along with Fourier-transform infrared spectroscopy were used with chemometric methods to investigate the change of primary and secondary metabolites of fig fruits belonging to 11 cultivars from different geographical origins and based on their peel chromatic coordinates. We also aimed to compare the throughput resolution of both fingerprinting techniques on fresh figs and to explore the associations between them. This research was performed in northern Morocco, which is one of the most important hotspots for fig cultivation and diversity worldwide [10]. This is the first attempt to understand the diversity of primary and secondary metabolites and their interactions in fresh fruits, as affected by the peel chromatic coordinates and the cultivars factors, within a comparative scheme of local and exotic fig trees planted in collection under the climate of northern Morocco, and using a set of analytical methods coupled with chemometrics.

## 2. Materials and Methods

### 2.1. Plant Material 

Fully ripened figs were randomly harvested during August and September of 2018. The samples consisted of 11 cultivars, including some cultivated in an ex situ collection, of which six were local clones and five exotic varieties (Table 1). The experimental design was a complete randomized block. Full maturity was determined when three-quarters of the fruit receptacles turned to reddish-purple and when the fig was easily detachable from the twig. Fresh mature figs were then measured for their chromaticity coordinates and afterward cut and frozen at −80 °C before lyophilization. Shortly afterward, lyophilized samples were grounded to a powder at room temperature using an IKA A11 Basic Grinder (St. Louis, MO, USA).

### 2.2. Growing Conditions 

The cultivars were planted as an ex situ collection in ferritic soil. During the maturity period, an average temperature of 27 °C was recorded alongside an average rainfall of 26.4 mm occurring in the last couple weeks of August. During the period between the third week of August and the first couple weeks of September, the fig collection received intense solar radiation. The ripening process was generally rapid, lasting several days from August to early September, with significant differences among cultivars (Table 1).

### 2.3. FTIR Fingerprinting

The FTIR fingerprinting was performed at room temperature, within the wavenumber range of 4000 and 450 cm^−1^ with 4 cm^−1^ as the spectral resolution level, using a Bruker Vertex 70 FTIR Spectrometer equipped with ATR accessory (Bruker Optics Inc., Ettlingen, Germany). Fig powder (100 mg) of each cultivar was analyzed in triplicate, of which every single IR spectrum corresponded to the accumulation of 32 scans. Prior to infrared (IR) spectra acquisition, an empty germanium crystal surface was recorded as a background and then systematically subtracted from each sample’s IR spectra. The germanium crystal was cleaned using technical ethanol between each measurement. For the infrared fingerprint assignments in Table 2, validation was obtained from standard assignments that have been detailed in the literature.

### 2.4. Fruit Peel Color

The chromatic coordinates were determined in the CIELab color space using a digital colorimeter (Minolta CM-700, Osaka, Japan). 

The coordinates measured were: lightness (L*), where negative values indicate darkness, while positive ones indicate lightness; a* (a coordinate reflecting the variation from redness to greenness); and b* (a coordinate describing the color variation between yellow and blue). The chroma (C*) coordinate indicates color intensity, whereas the hue angle (h°) predicts the visual color appearance, with 0° or 360° = red-purple, 90° = yellow, 180° = green, and 270° = blue. C* and h° were calculated as follow:

(1) $$h_{\mathrm{ab}}=\mathrm{arctg}\frac{b^{*}}{a^{*}}\to C_{\mathrm{ab}}^{*}=\sqrt{a^{*2\;}+b^{*2}\;}$$

Measurements were performed at two random positions around the equatorial region of each fruit with 15 replications for each genotype. The mean of the two measurements over a single fruit was assumed as one replicate. 

The present study focused particularly on L*, c* and h° indices, since a* and b* are merely coordinates that indirectly reflect hue and chroma.

### 2.5. HPLC Sugars and Organic Acids Profile

Chromatographic analysis of organic acids and sugars was carried out following Hernández et al. [20]. Thus, 0.5 g of lyophilized powder of each sample was diluted in 5 mL of Milli-Q water and then ultrasonicated (UP 400St (400 W, 24 kHz) for 30 min using Hielscher’s digital ultrasonicator. Shortly afterward, the solutions were centrifuged for 20 min at 15,000× *g* (Sigma 3–18 K; Sigma, Osterode am Harz, Germany). Sample extracts were then filtered using a 0.45 µm Millipore filter and kept in a dark room at a low temperature (4 °C) until HPLC analysis. Then, 10 µL of each sample’s hydrophilic extracts were pumped into a Hewlett-Packard HPLC (Series 1100, Wilmington, DE, USA) equipped with an autosampler, a refractive index detector (RID) and a diode array detector (DAD). The apparatus was also equipped with a precolumn (Supelguard, 5 cm × 4.6 mm; Supelco, Bellefonte, PA, USA) and then a column (Supelcogel TM C-610H, 30 cm × 7.8 mm) dedicated to the analysis of organic acids and sugars contained in the injected solutions. For the elution buffer, 0.1% phosphorus (pH = 3.0) was used at a flow rate of 0.5 mL min-1. Organic acids were detected at a wavelength of 210 nm by a diode array detector (DAD). The same analytical conditions were set for determination of the sugars profile using a refractive index detector. Standards (glucose, fructose, sucrose, citric acid and malic acid) for the abovementioned analyses were bought from Sigma (St. Louis, MO, USA). Calibration curves for both organic acids and sugars displayed good linearity (on average, r^2^ ≥ 0.999) within the ranges of 0.10 to 18.7 g L^−1^ and 0.031 to 4.8 g L^−1^ for sugars and organic acids, respectively. Results were expressed as g kg^−1^ of dry weight (dw).

### 2.6. Antioxidant Activity 

The antioxidant activity (AA) of the fig methanolic extracts (MeOH/water; 80/20%; *v/v*; +1% HCl) was assessed following two basic mechanisms. The first one investigated the free radical scavenging activity using two separate assays: the DPPH (radical 2,2-diphenyl-1-picrylhydrazyl) method as described by Brand-Williams et al. [21] at a wavelength of 515 nm and ABTS (2,2-azinobis-(3-ethylbenzothiazoline- 6-sulphonic acid)) following Re et al. [22] at 734 nm. The second mechanism aimed to assess the ferric reducing antioxidant power ability (FRAP assay) in the wavelength of 593 nm following Benzie & Strain [23]. The AA of all above assays was determined as mmol Trolox equivalent/g of dry weight (mmol TE/g dw).

Antioxidant capacity values were calculated using Equations (1) and (2):

I (%)_sample_ = [(Abs control − Abs sample)/Abs control] × 100 (2)

(3) $$\mathrm{mmol}\;\mathrm{TE}=\frac{\left(\left({I\%}_{\mathrm{sample}}-b\right)/a\right)\left(\mathrm{mg}/\mathrm{mL}\right)\times 10^{3}}{c_{\mathrm{sample}}\left(\mathrm{mg}/\mathrm{mL}\right){\times M}_{\mathrm{trolox}}\left(g/\mathrm{mol}\right)}$$

where Abs is the absorbance, a and b correspond respectively to the slope and the constant of the linear equation related to the standard curve of each essay, M_trolox_ is the molar mass of Trolox and C_sample_ is the sample concentration.

### 2.7. Total Polyphenols Analysis

First, polyphenol extracts were prepared by transferring 1 g aliquots of each powder into polypropylene tubes with 20 mL of ethanol solution 80% (80:20, *v/v* ultrapure water) at 4 °C for 15 min. The mixture was then centrifuged for 10 min at 3000× *g*, and the supernatant was separated from the pellet. The latter was homogenized and polyphenol extracts were removed as above, for a total of three extractions. The combined supernatants were then filtered through Whatman No.1 filter paper.

Total polyphenol content (TPC) was determined based on the Folin–Ciocalteu micro-method [24]. The method involved mixing 40 μL of phenolic extract of each sample, 3160 μL of Milli-Q water, 200 μL of the Folin–Ciocalteu reagent and 600 μL of sodium carbonate solution (20%). The mixtures were then incubated for 30 min at 40 °C, before their absorbances were measured at 765 nm (UV-1700 Shimadzu, Tokyo, Japan). TPC was expressed as mg Gallic acid equivalent (GAE) per 100 g of dry weight (dw).

### 2.8. Quality Assurance/Quality Control (QA/QC)

Method validation for the sugars and organic acids profiling was verified according to AOAC guidelines. The linearity of the HPLC described above was evaluated by analyzing the standard solutions used herein at different concentrations. An average correlation coefficient of 0.999 was obtained through calibration curves for all the standards for sugars and organic acids, respectively. Afterward, the recovery test was performed by spiking samples at different concentrations with known amounts of each standard. Spiked and unspiked extracts were then analyzed in triplicate. With regard to the complexity of the samples, satisfactory recovery levels were obtained (86 to 98% for reducing sugars and 89 to 97% for organic acids) alongside low standard error values within a narrow range of variation (0.07 to 1.23% for reducing sugars and 0.05 to 1.09% for organic acids). 

### 2.9. Statistical Analysis

Biochemical data were standardized to a comparable scale and then tested for their normality (µ = 0 and σ = 1) using SPSS v27. Data were then subjected to analysis of variance (ANOVA) to examine significant differences among the fig samples investigated herein. Differences in biochemical biomarkers across the studied cultivars were tested with Duncan’s new multiple range test (DMRT). Correlations among the studied variables were examined based on the Pearson model alongside their significance levels. 

FTIR spectra were corrected using the standard normal variate (SNV) along with multiplicative scattering correction (MSC) [25]. Afterward, the ATR correction procedure was applied as described by Hssaini et al. [26]. The corrected IR spectrum and the total integrated area were plotted for each sample using OriginPro software v9 in order to visualize differences in vibrational intensities among fig cultivars. Principal component analysis (PCA) was performed for both biochemical and FTIR data in order to determine the throughput resolution of discrimination among sampled cultivars using both approaches. A two-dimensional clustered heatmap (2D CHA heatmap) was then constructed following Ward’s method with the Euclidean distance using R software 3.0.2. The effect-size measure within the dataset was symbolized by the color gradient and intensity. The 2D CHA heatmap clusters similar rows and similar columns together based on the calculated similarity, which is represented by two dendrograms: one is sample-oriented while the second is variable-oriented. This method was applied to gain more insight into complex biological samples for which a one-way direction is most often expected [19].

**Figure 1:**
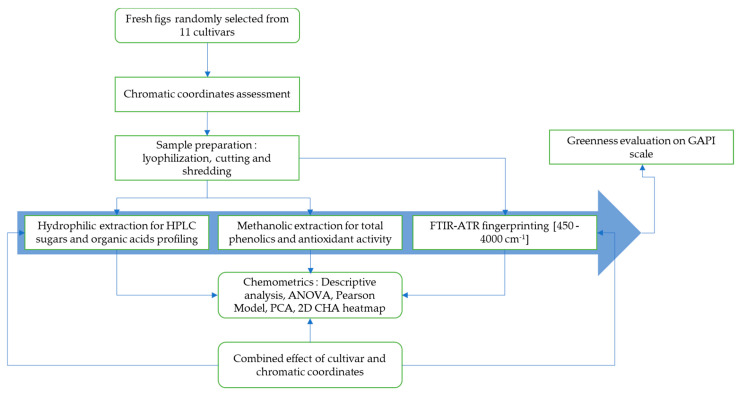
Flow chart of the steps involved in this study, showing how data were acquired and monitored to examine the effect of cultivar and peel chromaticity on primary and secondary metabolites. ANOVA: analysis of variance; PCA: principle component analysis; 2D CHA heatmap: two-dimensional clustered heatmap.

The schematic diagram below describes the processing steps applied in investigating the primary and secondary metabolites of the fresh figs sampled herein (Figure 1). A comparative scheme was used to compare methods such as HPLC—which is time-consuming, expensive and requires laborious work and an important number of chemical inputs, some of which are hazardous—and low-cost technologies, which are accurate, enable high-speed analysis and require minimal sample preparation, mainly vibrational spectroscopy (FTIR).

## 3. Results and Discussions

### 3.1. FTIR Fingerprinting 

**Figure 2:**
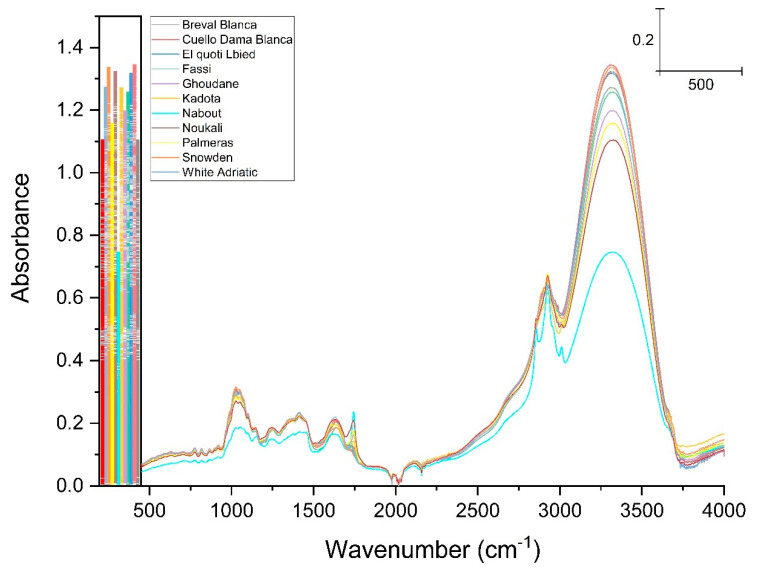
FTIR spectra of sampled figs within the wavenumber of 4500–450 cm^−1^.

**Figure 3:**
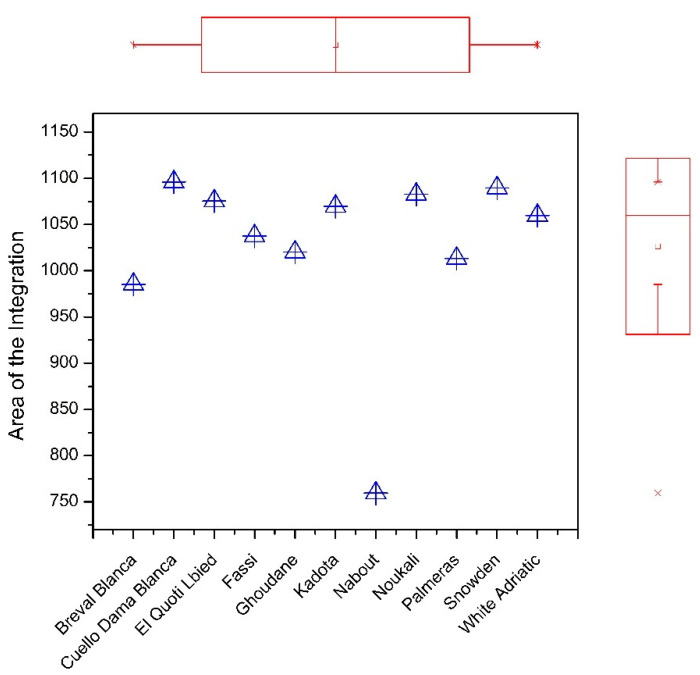
Marginal boxplot chart showing the total integrated area of the entire IR spectrum for each cultivar.

FTIR analysis displayed six major fingerprints with variable intensities of vibration (Figure 2); the assignment of each band is summarized in Table 2. The first peak at 3326 cm^−1^ was ascribed to the O–H stretching vibrations which result from hydrogen bonding in cellulose. This region recorded the highest absorbance and was most likely related to fibers that are highly present in fresh figs [27,28]. The vibration around the wavenumber of 2929 cm^−1^ was assigned to C–H, O–H and NH3 symmetric and asymmetric stretching, which is probably typical of carboxylic acids, carbohydrates and phenolics [29,30]. The sharp peak at 1745 cm^−1^ was associated with the vibration of the C=O of ester-type carboxylic [28,29,30]. The weak peak at 1630 cm was due to in-plane N–H bending and C–N stretching of amide I absorption, while the amide II vibration band most likely occurred around the very weak peak at 1414 cm^−1^, which displayed a less distinct mode of vibration [31]. The band in the region of 1414 and 1238 cm^−1^ corresponded to phosphodiester groups (Figure 2). This band was probably a result of several weak peaks that could not be differentiated among the analyzed samples. According to several studies, this band usually includes, among others, a vibration around 1392 cm^−1^, which is most likely related to carbohydrates, fatty acids or amino acid side chains [31,32]; the 1315 cm^−1^ vibration, which is associated with CH_2_ rocking [33]; and a band at 1230 cm^−1^, which is due to C–N elongation combined with N–H in-bending, alongside small vibrations from C–C elongation and C–O in-plane bending. This band was assigned to Amide III. Finally, there was a weak vibration around the 1155 cm^−1^ band, which was ascribed to C–O stretching [34]. At 1337 cm^−1^ there was a very distinct peak which can most likely be attributed to stretching of C–OH, C–C and C–O in the carbohydrate coupled with C–O stretching in phenolic compounds [35]. Since the acquired IR spectra generally overlapped, their full respective areas of integration were calculated and plotted in a marginal boxplot chart (Figure 3) in order to visualize the differences among the sampled cultivars based on their vibration intensities. Figure 3 displays significant differences across samples, for which the IR integrated areas ranged between 985 for ”Breval Blanca” and 1095 for “Cuello Dama Blanca”. Obviously, the cultivar ”Nabout” was distinguished from the other cultivars as it had the lowest integrated area (759). Clearly, the results herein presented suggest that the FTIR-ATR fingerprinting technique could be accommodated as an accurate and nondestructive method allowing high-speed analysis for investigation of fig structural characteristics and functional properties. 

### 3.2. Peel Color 

**Figure 4:**
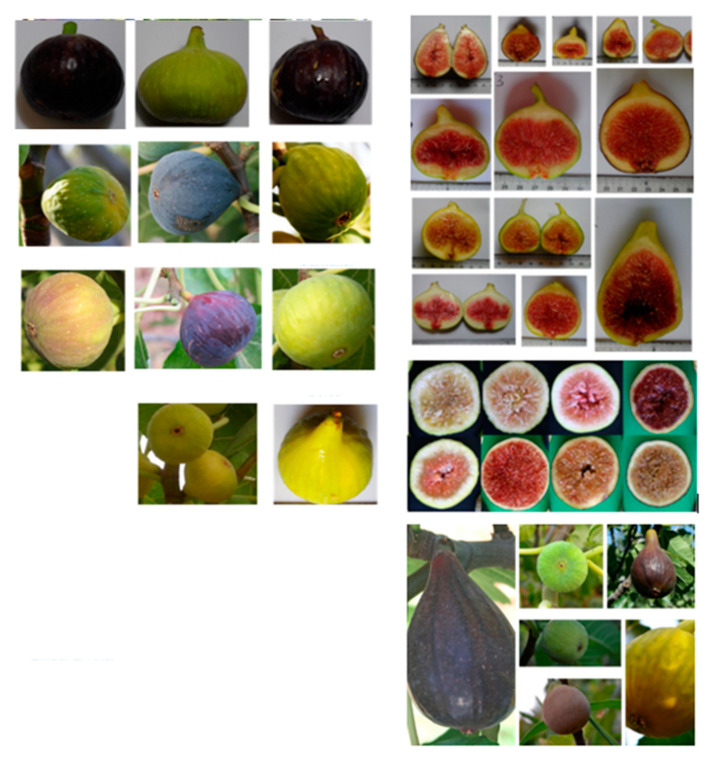
Image showing the diversity in fig chromatic coordinates for both peel and receptacle.

The fig peel color showed a high significant variability between cultivars (*p* < 0.001) (Table 3). Hence, it varied from a bright green-yellow color (high and positive values of L* and c*) to an atypical dark purple color (negative L* and c* values and high hue values), with manifestation of some intermediate colors (brown, red purple, blue-purple, etc.). Lightness (L*) ranged from 31.72 ± 3.75 to 83.64 ± 5.37, recorded by “Fassi” and “Palmeras”, respectively. These cultivars correspondingly displayed the lowest and the highest chroma values (10.28 ± 0.46 and 42.12 ± 7.67, respectively). Indeed, the cultivars “Palmeras”, “Nabout”, “Breval Blanca” and “Cuello Dama Blanca” appeared to have the brightest fruit peel colors, whereas “Fassi”, “Noukali” and “Ghoudan” had the darkest colored figs. Cultivars with light-colored figs had a hue angle (h°) oscillating between 95.72° ± 9.06 (“El Quoti Lbied PS20”) and 115.7° ± 15.42 (“White Adriatic”), which is in the range of the green–yellow colors. However, those with dark colored figs recorded h° in the range of 205° and 360°, showing blue to red-purple colors (Table 3). Generally, figs display large scale variability with regard to their peel and receptacle colors, particularly driven by the genetic factor, as seen in the Figure 4. According to the literature, the fruit color coordinates are generally correlated to the flavonoid content, typically to anthocyanins [6]. Therefore, peel color evaluation using these coordinates is of great importance in vegetable quality assessment. Several studies have focused on exploring potential correlations between chromatic coordinates and antioxidant compounds, essentially phenols (anthocanins, tanins, catechins, etc.) and carotenoids (lycopene, beta-carotene, etc.) [10,11,12,13,14,15,16,17]. Our results, in terms of the range of variation of fruit peel color, were in accordance with those of Viuda-Martos et al. [36] and Sedaghat & Rahemi [37].

### 3.3. Reducing Sugars

Individual sugars showed significant differences amongst cultivars (*p* < 0.001), where glucose (GLUC) and fructose (FRUC) concentrations varied within similar ranges. Hence, the glucose/fructose ratio ranged between 0.85 and 1.06. Nevertheless, only minor concentrations of sucrose were found. Glucose amounts varied between 5.55 ± 0.27 g kg^−1^ dw (“Ghoudan”) and 29.94 ± 0.81 g kg^−1^ dw (“Breval Blanca”), whereas fructose concentrations were in the range from 6.29 ± 0.28 (“Ghoudan”) to 26.15 ± 0.78 g kg^−1^ dw (“Breval Blanca”). It is noteworthy that the local cultivars “Fassi”, “Ghoudan” and “Noukali”, along with the introduced varieties “Palmeras” and “White Adriatic”, exhibited higher fructose levels compared to glucose, while the other cultivars displayed the inverse tendency (Table 4). These results show the genetic influence on the glucose/fructose ratio in fig cultivars. According to [38], this aspect is of great importance, since fructose is 80% sweeter than sucrose, whereas glucose is only about 60% sweeter than sucrose. Accordingly, figs exhibiting a lower glucose/fructose ratio are assumed to display a higher degree of sweetness in comparison with other cultivars. Regarding fructose, concentrations varied sensitively among the studied samples from 6.23 ± 0.28 g.kg^−1^ dw to 28.15 ± 0.78 g kg^−1^ dw, recorded by “Ghoudan” and “Breval Blanca”, respectively (Table 4). These values of glucose and fructose confirm the findings reported in several studies [12,14,38,39]. All cultivars showed low amounts of sucrose (SUCR) that varied between 0.86 ± 0.01–3.66 ± 0.28 g kg^−1^ dw (Table 4). This was due to anabolic processes and respiration during fruit development. It might also have been caused by the hydrolysis of sucrose into fructose and glucose during fruit ripening [12,13,14,15].

The cultivar “Breval Blanca” was particularly rich in reducing sugars as it contained 59.28 g kg^−1^ dw, followed by “Palmeras” with 48.64 g kg^−1^ dw, whereas, “Ghoudan” contained the lowest amounts of total sugars (12.64 g kg^−1^ dw) (Table 4). Generally, the amounts of figs’ reducing sugars are comparable to those reported in some other fruits, such as apples and strawberries [17,18,19,20,21,22,23,24,25,26,27,28,29,30,31,32,33,34,35,36,37,38,39,40]. Clearly, since the sweetness is an important factor that particularly contributes to the figs’ taste quality and is one of the most important selection criteria in the breeding programs of this species, cultivars with higher values of reducing sugars and the lowest value for the glucose/fructose ratio should be promoted.

### 3.4. Organic Acids

**Figure 5:**
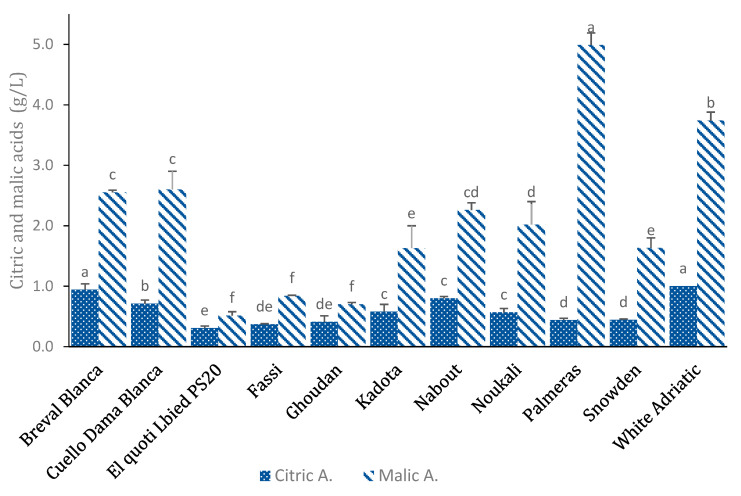
Variations in organic acids contents (g kg^−1^) among the studied cultivars (average values ± standard deviation). Letters (a–f) over error bars (a–f) denote statistically significant differences based on one-way ANOVA.

The organic acids contents in the studied cultivars are given in Figure 5. Results showed high significant differences among the studied cultivars. Malic acid was the most abundant organic acid in all samples and was recorded in very high levels compared to citric acid. The Spanish cultivars “Palmeras”, “Cuello Dama Blanca” and “Breval Blanca” exhibited the highest malic acid concentrations, with the average values being 4.99 ± 0.2, 2.6 ± 0.3 and 2.55 ± 0.04 g kg^−1^ dw, respectively (Figure 5). Similarly, important variability was detected for citric acid amounts, with values ranging from 1 ± 0.02 (“White Adriatic”) to 0.31 ± 0.04 (“El Quoti Lbied PS20”) (Figure 5). These results are in agreement with those reported by Colaric et al. [41], Veberic et al. [9], Pande & Akoh [16] and Pereira et al. [42]. The same authors reported that both malic acid and citric acid were identified as the main fig organic acids. Nevertheless, oxalic, fumaric, succinic and shikimic acids were also identified but in minor levels.

In addition to sugar amounts, fruit sweetness is also correlated to certain acids, such as citric and shikimic acids. In peaches, citric acid can, even in small amounts, increase the sourness of the fruit more than malic acid. Moreover, despite high sugar levels, the presence of some particular organic acids, even in minor amounts, can influence the taste of fruit and decrease their sweetness [41]. Compared to some fruits, such as sweet cherries, peaches or apricots, figs present lower amounts of citric and malic acids [41,42,43].

### 3.5. Sugars/Organic Acids Ratio

**Figure 6:**
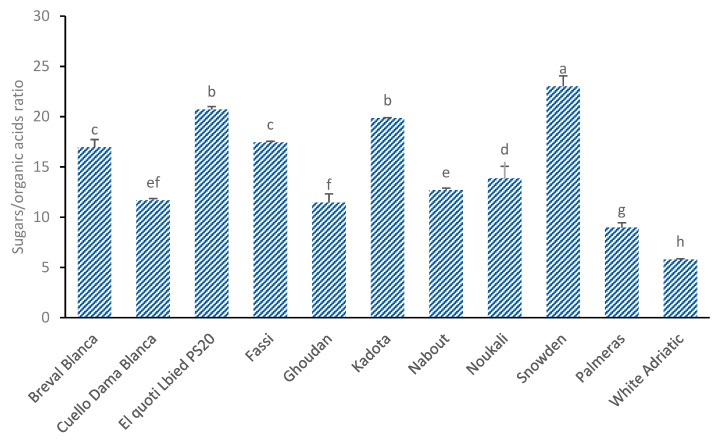
Sugars/organic acids ratio in the 11 studied cultivars of fig fruit. Average values ± standard deviation are presented; statistically significant differences among cultivars are displayed over error bars (a–h).

Like the glucose/fructose index that indicates the fig sweetness, the ratio between the total sugars and organic acids in figs at full maturity (Figure 6) is also an important quality index and a good indicator of fruit quality and flavor [43]. The higher the ratio, the sweeter the fruits are, and the lower the ratio, the more bitter they are [41]. There were significant differences among cultivars for the sugars/organic acids ratio (*p* < 0.01). Although sugars are important in fig cultivars, organic acids, even at minor levels, have an important influence on the taste of fruit and decrease their sweetness [41]. The highest value for the sugars/organic acids ratio was found in the American variety “Snowden” (20 ± 1.05) due to its having the highest content of sugars and an average level of organic acids. The local cultivar “El Quoti Lbied” recorded the lowest content of organic acids and optimal total sugars and thus had a high ratio level (20.7 ± 0.28). However, the lowest ratio levels were found in “Palmeras” (8.96 ± 0.47), “Ghoudan” (11.45 ± 0.86) and “Cuello Dama Blanca” (11.67 ± 0.17) (Figure 6).

### 3.6. Total Phenol

**Figure 7:**
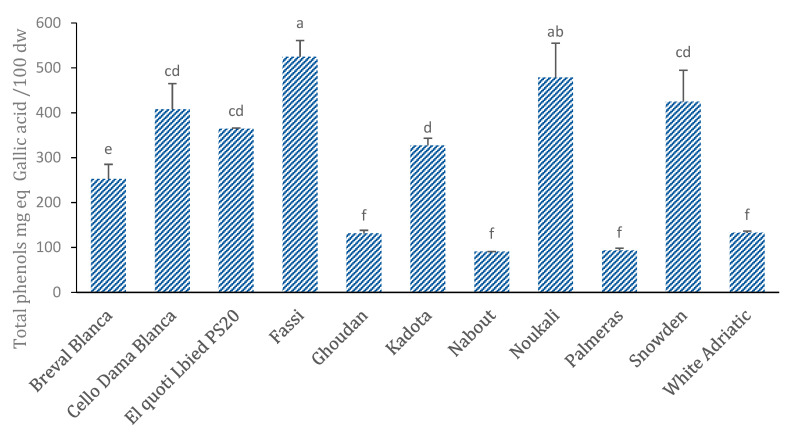
Total phenolic content (mean ± SE in mg GAE/100 g dw) of the whole fruit for 11 fig cultivars. Average values ± standard deviation are presented. Statistically significant differences among cultivars are presented over error bars (a–f).

Measurement of the total phenol (TP) contents revealed highly significant variability among the cultivars (Figure 7). The cultivar “Fassi” exhibited the highest amount of TP (524.74 ± 35.9 mg GAE/100 g dw) followed by “Noukali” and “Snowden” (478.64 ± 76.12 and 42.52 ± 69.98 9 mg GAE/100 g dw, respectively). The lowest amounts were found in “Nabout”, “Palmeras” and “Ghoudan”, where the average values were respectively 90.75 ± 0.05, 93.35 ± 4.85 and 131.21 ± 6.9 mg GAE/100 g dw (Figure 7). The findings were similar to those reported in previous works [10,13,44,45]. However, they were lower than those of other studies on commercialized figs [3,11,46]. Clearly, cultivars with dark-colored figs have high amounts of TP compared to cultivars with light-colored ones. Similar results have been reported in several studies [1,5,11].

According to the literature, the total phenol content is the main contributor to the plants’ antioxidant capacity, but it also contributes to their bitter, sweet or astringent flavors alongside sugars and organic acids. Furthermore, phenols typically influence the aroma profile [1]. It is generally acknowledged that phenols are mainly concentrated in fig peels, as well as in other fruits such as apricots and apples [5].

### 3.7. Antioxidant Activity 

Results for the antioxidant activity (AA) based on the radical scavenging activity (DPPH and ABTS) and ferric reducing ability (FRAP) are summarized in Table 5. Highly significant variability (*p* < 0.001) was found among cultivars with regard to these assays. In the DPPH assay, the highest AA was recorded for “Nabout” and “Breval Blanca”, for which the respective values were 88.1 ± 3.37 and 83.16 ± 6.93 mmol TE eq/g dw, whereas the lowest potentials were exhibited by “El Quoti Lbied” (14.28 ± 1.42 mmol TE eq/g dw) and “Snowden” (14.76 ± 1.61 mmol TE eq/g dw) (Table 5).

For the ABTS assay, the highest value was recorded for “Noukali” (8.04 ± 0.3 mmol TE eq/g dw), followed by “Breval Blanca” and “Fassi” (6.41 ± 0.66 and 5.96 ± 0.52 mmol TE eq/g dw, respectively). However, the lowest concentrations did not exceed 1.8 mmol TE/g dw and these were recorded by “Palmeras”, “Snowden” and “Ghoudan”, for which the average concentrations were 1.43 ± 0.25, 1.75 ± 0.51 and 1.76 ± 0.48 mmol TE eq/g dw, respectively. The local cultivars “Nabout” and “Noukali” showed the highest ferric reducing ability, with average values of 10.65 ± 0.18 and 7.44 ± 2.2 mmol TE eq/g dw, respectively. The lowest ferric reducing abilities were recorded by light-colored cultivars, mainly “El Quoti Lbied” and “White Adriatic” (1.09 ± 0.04 and 1.97 ± 0.88 mmol TE eq/g dw, respectively) (Table 5). In contrast to the scavenging potential revealed by the DPPH and ABTS assays, which showed that light-colored cultivars contained the highest values, the ferric reducing capacity was greater in dark-colored figs. For ABTS plus the radical scavenging activity and ferric reducing ability, the results are consistent with those of Veberic & Mikulic-Petkovsek [1], Solomon et al. [5] and Ercisli et al. [6]. However, DPPH radical scavenging capacity results were higher than those described in similar studies [6,9,14,16].

Generally, most of the cultivars that showed the highest potential for ferric reducing activity and free radicals’ inhibition recorded significant levels of total phenols. Moreover, in contrast with findings reported in previous studies, cultivars with black and purple peels had lower total antioxidant capacities than light-colored cultivars [5,11,13]. This may be referred to the differences in the amounts of anthocyanins, particularly cyanidin-3-rutinoside, which was found to be the major contributor to the antioxidant capacity of the investigated fig fruits [5,6,7,8,9,10,11,12,13,14,15,16,17,18,19,20,21,22,23,24,25,26,27,28,29,30,31,32,33,34,35,36,37,38,39,40,41,42,43,44,45,46,47]. 

### 3.8. Correlations Among Variables

The bivariate correlations of all variables used to evaluate the fig accessions were considered using the Pearson coefficient in order to analyze the relationships between the evaluated fig attributes.

Only significant correlations at the 0.01 level with coefficients of correlation greater than or equal to |0.5| were considered as being important. Citric acid level was positively correlated to sucrose amounts (r = 0.518, *p* < 0.01) (Table 6). The latter is the main translocation carbohydrate from leaves to other plant parts, particularly to the fruit. It particularly contributes, along with citric acid, to the sweetness of the fruit. Similarly, fructose was strongly correlated to malic acid (0.583, *p* < 0.01), which means that sucrose and malic acid increase during fig ripening while sucrose decreases by invertase activity, which gives to the fruit a higher sweetness at its full maturity. Citric acid levels eventually decrease, as do those of sucrose, which explains the high levels of malic acid and minor amounts of sucrose in all samples at their full maturity. Malic acid was also positively correlated to the lightness and the chroma coordinates (r = 0.734, *p* < 0.01 and r = 0.499, *p* < 0.01, respectively). Similarly, glucose and fructose were positively correlated to the lightness coordinate color L* (r = 0.496, *p* < 0.01 and r = 0.548, *p* < 0.01 respectively), which means that cultivars with light-colored fruits contain higher levels of malic acid and reducing sugars (Table 6). This means that light-colored fruits were the sweetest among studied samples. This makes sense, since the ratio between sugars and organic acids was generally higher in fruits displaying high levels of chroma and lightness. Among the antioxidant assays, the DPPH radical scavenging capacity test showed important and positive correlations with malic acid alongside reducing sugars glucose and fructose (r = 0.524, *p* < 0.01; r = 0.473, *p* < 0.01; and r = 0.455, *p* < 0.01, respectively) (Table 6). These correlations have been previously attributed to the role of carbohydrates as genuine ˙OH scavengers due to the H atom cation from molecules of reducing sugars. The results reported here confirmed the findings reported in previous work in which antioxidant capacity was found to be strongly and positively correlated to the total phenol content and, particularly, to anthocyanins, which have been revealed as the major contributors in figs’ radical scavenging activity [5,11,13].

The findings herein reported are undoubtedly of particular importance for fig selection and future breeding programs. In fact, these biomarkers can be exploited in further studies as they can predict each other following the significance level, strength and direction of their associations. Examination of potential correlations in the dataset may provide important insights into the variables most relevant for fig genotype assessment and classification. In this sense, statistically significant and strongly linked traits, like peel chromatic coordinates, could be exploited to predict other traits, particularly antioxidant compound levels, and recognized as of significant importance for assessment, discrimination and classification of fig genotypes, alongside orienting industrial activity.

### 3.9. Chemometric Analyses 

Chemometrics and data imaging are essential tools for understanding datasets of biological samples. Methods such as principal component analysis (PCA) are usually used to determine variables that capture the high variance within a dataset. A dispersion of observations, called a scatter-plot, can be drawn using dimensionality reduction onto two- or three-dimensional spaces [48]. This unsupervised variable construction method was applied to both biochemical and FTIR fingerprinting to compare their throughput resolution of discrimination.

However, this transition is costly, often resulting in loss of the total variance. Thus, two-dimensional clustered heatmaps were produced for biochemical data, as these do not need a dimensionality reduction to visualize data and display network connections in a symmetric adjacency matrix [48]. In the present study, PCA, being an unsupervised variable construction method, was used for both biochemical and FTIR fingerprinting to compare their throughput resolution of discrimination, while heatmaps were used for biochemical data to cluster cultivars, based on the correlation matrix, according to peel color and cultivar origin.

#### 3.9.1. Principal Component Analysis

##### FTIR Fingerprinting

**Figure 8:**
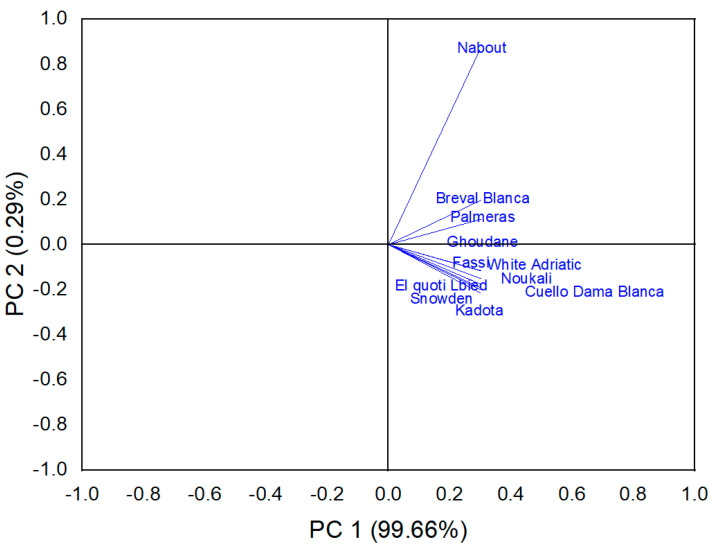
Score plot of the PCA of the FTIR-ATR spectra within the wavelength number range of 4000–450 cm^−1^.

A PCA model based on the FTIR spectroscopy data for the entire wavenumber range (450–4500 cm^−1^) revealed two major clusters, with a distinctive classification for the cultivar ”Nabout”, which displayed the lowest vibration intensities among all the studied cultivars (Figure 8). The first cluster regrouped “Breval Blanca” with “Palmeras”, while the second included the rest of the cultivars. These two clusters were discriminated based on the total area of intensity within the entire range of vibration. Thus, the first cluster had the lowest integrated intensity: 985 and 1013, respectively, for “Breval Blanca” with “Palmeras”. The second group yielded the highest integrated intensity, ranging between 1020 and 1089. 

**Figure 9:**
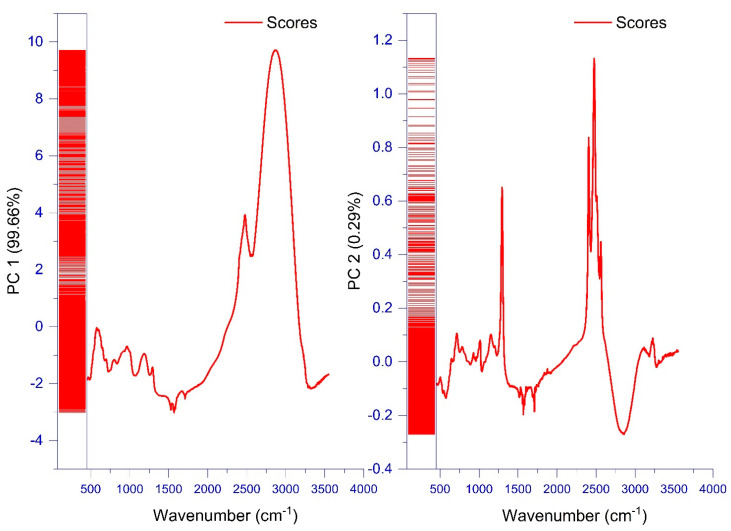
Loadings of each wavelength number in the PCA model total variance for the investigated fig samples.

**Figure 10:**
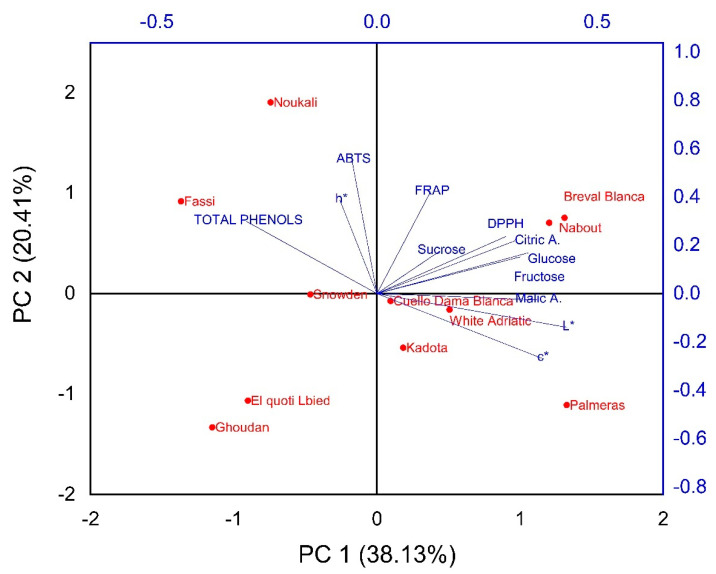
Biplot of scores and loadings for the PCA, constructed based on biochemical screening.

The vibration in the region of 3000–2800 cm^−1^ and the peak at 2500 cm^−1^ captured the highest variance explained by the model. These two distinct and large peaks were associated with C-H, O-H and NH_3_ and CH2 stretches, respectively. They were attributed to carbohydrates, phenols and free amino acids along with the hydroxyl group of carboxylic acid, respectively (Figure 9). This result makes sense, as these compounds are among the major biochemical components in fresh figs [7,8,10]. 

##### Biochemical Screening

In order to determine discriminant variables in the dataset, principal component analysis based on the correlation coefficient was performed. This analysis aimed to define the main factors that contribute to fig trees’ discrimination. In our study, only a principal component loading of more than |0.5| was considered as being significant for each factor. Total variance of 70% was explained by the first three components (Table 7). 

The first component consisted of eight variables, citric and malic acids, glucose, fructose, total phenol, DPPH free radical scavenging, lightness L* and chroma C*, for which the scores were respectively 0.672, 0.797, 0.717, 0.764, 0.587, −0.568, 0.871 and 0.760. It explained about 36% of the total variance observed, which means that these attributes had the highest impact on their discrimination (Table 6). The second component accounted for about 20% of total variance and was defined by ABTS plus radicals scavenging and ferric reducing ability, for which the respective scores were 0.853 and 0.694. The third component accounted for 14.15% of total inertia and was mainly correlated to the amount of sucrose (0.856) and the hue angle h° (0.626) (Table 6).

The biplot in Figure 10 was built based on the first two components and displays large numbers of clusters compared to those revealed by FTIR spectroscopy. However, some similarities can be spotted between both fingerprinting techniques. Thus, the cultivars “Breval Blanca” and “Nabout” were clustered as distinctive subclusters; furthermore, “Palmeras” was also classified as a single item. These two subsets were contrasted based on the initial components (PC1), which in particular explained the chromaticity and organic acids and sugars content. Even though both subsets had light-colored figs, they displayed contrasting levels in sugars and organic acids concentrations. Thus, “Palmeras” had low glucose and fructose levels compared to “Breval Blanca” and “Nabout”, alongside a high malic acid content compared to the same cultivars. It is noteworthy that these cultivars were largely distinguished from other samples. Furthermore, “Noukali” and “Fassi” were classified as a distinctive subset, despite having contrasting peel colors. Likewise, “El Quoti Lbied” and “Ghoudan” had contrasting peel chromaticity but were clustered as a homogenous subgroup, which was due to their similar antioxidant potency.

The slight dissimilarity regarding the other cultivars was due to the fact that the biochemical variables herein investigated alongside chromaticity could not totally explain the cultivars’ classifications based on FTIR spectroscopy. The same point was reported by Harvey et al. [49]. Overall, the discrimination throughput resolution offered by both approaches suggests that FTIR spectroscopy for fig screening and discrimination based on primary and secondary metabolites is a cost-effective, rapid and low-cost technology that is accurate with minimal sample preparation, as well as being environmentally friendly in comparison to wet chemical methods.

#### 3.9.2. Two-Dimensional Clustered Heatmap

**Figure 11:**
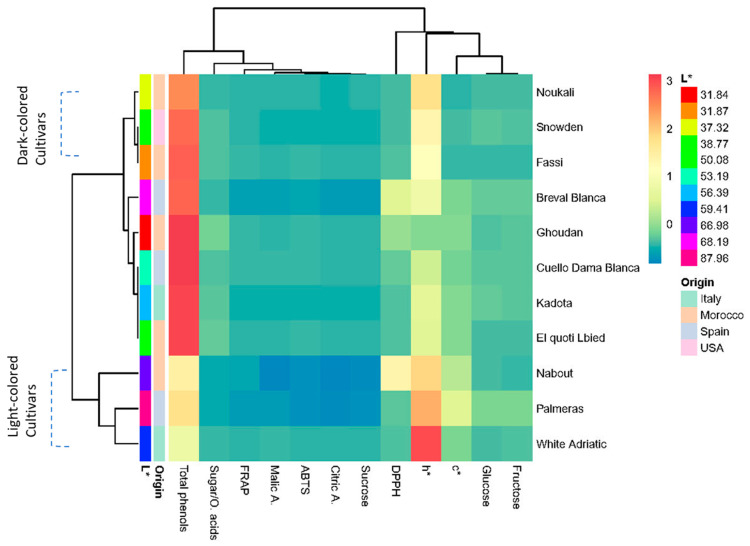
Two-dimensional hierarchically clustered heatmap based on the correlation matrix of the studied variables.

A color-coded two-dimensional heatmap for the biochemical attributes of all cultivars was constructed with two clusters, using Euclidean distance and following the Ward method. One cluster was sample-oriented whereas the other was variable-oriented (Figure 11). Figure 11 shows a data matrix, where coloring gives an overview of the numeric differences. In this figure, weak correlations between studied variables are displayed with low color intensity, while stronger ones are shown with high color intensity. The heatmap showed that the chromatic coordinates, mainly the hue angle and total phenol, had the higher scores in the dataset, which means that they had the higher effect in cultivar clustering. However, the other variables showed a very weak impact. The figure yielded a combined dendrogram which was clearly differentiated into two main clusters. The first one was subdivided into two subsets where dark-colored cultivars, including “Noukali”, “Snowden” and “Fassi”, were grouped, while their nearest neighbors, “Cuello Dama Blanca”, “Breval Blanca”, “Kadota” and “El Quoti Lbied” had light-colored figs with L* < 60. The second cluster included three cultivars with very light-colored peels. These cultivars were “Nabout”, “Palmeras” and “White Adriatic”. It is noteworthy that, contrary to the peel color, the geographical origin of these cultivars presumably did not have any clear impact on their clustering. Finally, both the PCA (unsupervised method) and heatmap (supervised variable construction method) helped to better understand the relationship between the variables and resulting clusters. The slight differences between the two statistical methods were due to their respective data processing procedures.

### 3.10. Evaluation of Greenness of the Analytical Techniques Used on the Scale of the Green Analytical Procedure Index (GAPI)

**Figure 12:**
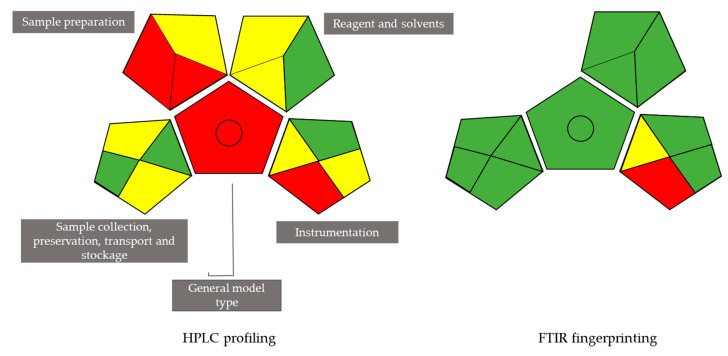
Green Analytical Procedure Index (GAPI) pictogram for ecological impact evaluation of analytical methods used in this study.

Analytical techniques used for biological matrix assessment are mostly complex and involve numerous steps. Thus, examination of their overall ecological impact may not be easy. Therefore, a number of tools are currently advocated to evaluate and compare different techniques in terms of their greenness [50,51]. The Green Analytical Procedure Index (GAPI) is one of the greenness evaluation methods that has gained particular interest as it covers the entire analytical pathway, as compared to other, earlier analytical eco-scales, such as the National Environmental Method Index (NEMI) [52]. The GAPI evaluates fifteen aspects of the analytical method and is composed of five pentagrams. Each one describes a single step in the analysis procedure, including sample preparation (transportation, storage and preparation), the chemical inputs involved and instrumentation, alongside the method purpose [51]. The index is based on a color-code scale, where red symbolizes high hazard to environment and yellow and green represent lower impacts with better tolerability with respect to environment. Further detail regarding this approach can be found in the paper by Kurowska-Susdorf et al. [53]. Figure 12 displays the GAPI for the two analytical techniques (HPLC and FTIR) used in this article. Thus, the HPLC GAPI pictogram displays many red sections within its pentagrams compared to only one in that of the FTIR technique. It is noteworthy that the FTIR GAPI pictogram is missing one pentagram, since this method does not require sample preparation. Besides displaying a red general model type, the HPLC GAPI showed only four green sections, while that of FTIR had only one yellow section. This is due to the fact that chromatographic techniques consume chemicals, some of which are hazardous and require particular sample preparation, while FTIR is an ecofriendly method that does not require chemical inputs [26].

## 4. Conclusions

The use of chemometric approaches to investigate the associations of chemical fingerprints within fig cultivars based on biochemical and FTIR screening was an attempt to provide a comprehensible tool for better understanding of complex biological systems where a one-way direction is assumed. Fourier-transform infrared spectroscopy fingerprinting in the wavenumber range of 450 and 4500 cm^−1^ displayed six major peaks, with the highest vibration intensity recorded around 3326 cm^−1^. The results for the peel color antioxidant activity and primary and secondary metabolites analysis of the 11 fig cultivars (*Ficus carica* L.) confirmed that fig species are an important source of natural bioactive compounds. This study reported some important findings regarding internal quality parameters in fig fruits and varietal effects. At full maturity, malic acid was the predominant organic acid in all cultivars. Although fructose and glucose contents had almost the same levels across all cultivars, glucose was found to be predominant in some cultivars, while fructose was more abundant in others. However, only minor amounts of sucrose were measured. Free radical scavenging investigated using the DPPH assay exhibited the highest antioxidant activity. The latter was significantly important in light-colored figs. Statistical analysis showed a wide variation in all the evaluated parameters that are very important for fig breeding. The results also showed that malic acid and reducing sugars (fructose and glucose) were positively correlated with the lightness and the chroma coordinate, whereas the total phenol content was significantly higher in dark-colored figs. Chemometric analysis using PCA for both biochemical and FTIR fingerprinting displayed satisfactory throughput discrimination, with slight dissimilarity attributed to the fact that biochemical attributes alongside in vitro antioxidant activity and chromaticity could not totally explain the cultivar classification as revealed by FTIR spectroscopy, where the total variance explained was higher. Color-coded visualization of the clustered data via dendrograms and heatmaps was of great use to identify the most discriminant biochemical variables and to understand their changes among the studied cultivars based on peel color and the cultivar origin. Furthermore, the greenness evaluation, using the GAPI scale, of the analytical methods used herein showed that the vibrational spectroscopy technique is more ecofriendly compared to chromatographic methods. Thus, a mutual usage of both techniques can be envisaged to mitigate the hazards of extensive toxic solvents and energy use. This approach can be considered an affordable methodology, but one of the limitations of this work was that a larger sample length was required and further validation should be performed using samples from other varieties and origins.

## Figures and Tables

**Table 1:** Cultivars’ geographical origins and harvest time and monthly meteorological data from August to early September 2018 for northern Morocco, Meknes (Ain-Taoujdate experimental station—INRA).

	Cultivar	Geographical Origin	August						September	
			(1–5)	(6–10)	(11–15)	(16–20)	(21–25)	(26–30)	(31–4)	(5–9)
Local	El Quoti Lbied	Morocco								
	Nabout									
	Fassi									
	Noukali									
	Ghoudan									
Introduced	Snowden	USA								
	White Adriatic	Italy								
	Kadota	Italy								
	Cuello Dama Blanca	Spain								
	Breval Blanca	Spain								
	Palmeras	Spain								
Total rainfall (mm)			0	0	0	0	0	26.4	0	0
Average temperature (°C)			25.84	28.5	27.56	29.24	29.44	23.64	25.6	25.42
Average solar radiation (W/m^2^)			169.29	208.74	243.83	238.28	185.35	123.5	270.21	271.38
Soil type			Sandy clay loam with an average organic matter of 1% (0–30 cm soil layer)							
Soil pH			7.2							

Climatic data collected from meteorological station installed next to the orchard. Texture organic matter was assessed with a composite sample using the Walkley–Black and Robinson methods respectively. The blue color describes the maturity period of each fig trees herein investigated in term of number of days (each column represents five days).

**Table 2:** IR assignments of the main vibrations in the FTIR spectra.

Wavenumbers (cm^−1^)	Assignment	References
3326	Intra-molecular hydrogen bonding C(3)OH···O(5) C(6)O···(O)H	[27,28]
2929	C-H symmetric and asymmetric stretching of CH3 and CH2 groups	[29,30]
1745	Stretching of C=O	[28,29,30]
1630	N–H bending and C–N stretching of amide I	[30,31]
1414 and 1238	N-H in-bending and C–N stretching of amid II CH, CH2 and CH3 deformations	[32,33,34]
1025	Stretching of C–OH, C–C and C–O	[34,35]

**Table 3:** Skin coordinate colors of studied cultivars’ fruits.

Cultivar	L*	c*	h*
Breval Blanca	63.92 ± 6.92 ^b^	36.34 ± 3.48 ^cd^	103.55 ± 3.23 ^cd^
Cuello Dama Blanca	60.26 ± 16.53 ^bc^	33.44 ± 2.39 ^d^	100.14 ± 4.82 ^cd^
El Quoti Lbied	48.8 ± 1.79 ^d^	37.29 ± 1.13 ^bc^	95.72 ± 9.06 ^cd^
Fassi	31.78 ± 3.75 ^f^	10.28 ± 0.46 ^f^	215.93 ± 16.81 ^c^
Ghoudan	34.91 ± 6.32 ^ef^	15.83 ± 2.99 ^e^	19.24 ± 14.93 ^d^
Kadota	56.12 ± 7 ^c^	37.44 ± 4.62 ^bc^	106.47 ± 5.66 ^cd^
Nabout	64.03 ± 3.19 ^b^	40.04 ± 3.96 ^ab^	105.2 ± 3.48 ^cd^
Noukali	32.43 ± 11.87 ^f^	6.2 ± 0.37 ^g^	356.14 ± 12.85 ^b^
Palmeras	83.64 ± 5.37 ^a^	42.12 ± 7.67 ^a^	102.63 ± 7.43 ^cd^
Snowden	39.8 ± 1.48 ^ef^	17.71 ± 0.59 ^e^	185.47 ± 19.81 ^c^
White Adriatic	60.49 ± 3.43 ^bc^	36.08 ± 1.16 ^cd^	115.7 ± 15.42 ^a^
Mean	51.23 ± 6.07	27.64 ± 2.54	181.47 ± 11.03
ANOVA mean square	2686.33 ***	1725.34 ***	215052.33 ***

Average values ± standard errors of the mean are presented. Different letters (a–g) in columns represent statistically significant differences among cultivars at *p* < 0.05; *** denotes significant differences at the level of *p* < 0.001.

**Table 4:** Content levels of individual sugars in the whole fruit for 11 fig cultivars.

Cultivar	SUCR (g kg^−1^ dw)	GLUC (g kg^−1^ dw)	FRUC (g kg^−1^ dw)	Total Reducing Sugars (g kg^−1^ dw)
Breval Blanca	1.19 ± 0.07 ^f^	29.94 ± 0.81 ^a^	28.15 ± 0.78 ^a^	59.28 ^a^
CuelloDama Blanca	1.68 ± 0.05 ^e^	18.52 ± 2.4 ^de^	18.47 ± 2.34 ^d^	38.67 ^d^
El Quoti Lbied	1.61 ± 0.25 ^e^	7.82 ± 1.05 ^fg^	7.57 ± 1.01 ^fg^	17 ^h^
Fassi	2.03 ± 0.06 ^cd^	9.48 ± 0.18 ^f^	9.6 ± 0.26 ^f^	21.11 ^g^
Ghoudan	0.86 ± 0.01 ^g^	5.55 ± 0.27 ^g^	6.23 ± 0.28 ^g^	12.63 ^i^
Kadota	2.36 ± 0.34 ^c^	21.41 ± 4.8 ^cd^	20.11 ± 4.5 ^cd^	43.88 ^c^
Nabout	2.93 ± 0.14 ^b^	18.46 ± 0.62 ^de^	17.4 ± 0.51 ^d^	38.79 ^d^
Noukali	2.25 ± 0.07 ^c^	16 ± 2.16 ^e^	17.23 ± 0.86 ^d^	35.47 ^e^
Palmeras	1.80 ± 0.31 ^de^	23.29 ± 0.35 ^bc^	23.55 ± 0.41 ^b^	48.63 ^b^
Snowden	1.13 ± 0.04 ^fg^	24.68 ± 1.01 ^b^	21.85 ± 0.9 ^bc^	47.65 ^b^
White Adriatic	3.66 ± 0.28 ^a^	10.98 ± 0.1 ^f^	12.85 ± 0.61 ^e^	27.49 ^f^
Mean	1.95 ± 0.15	16.92 ± 1.25	16.64 ± 1.13	17 ^h^
ANOVA mean square	2.04 ***	179.61 ***	143.47 ***	

Average values ± standard errors of the mean are presented. Different letters (a–i) in columns represent statistically significant differences among cultivars at *p <* 0.05. SUCR: sucrose; GLUC: glucose; FRUC: fructose. *** denotes significant differences at the level of *p* < 0.001.

**Table 5:** Free radical scavenging activity (DPPH and ABTS) and ferric reducing ability (FRAP) (mean ± SE in mmol TE eq/g dw) of the studied cultivars.

Cultivar	DPPH (mmol TE eq/g dw)	ABTS (mmol TE eq/g dw)	FRAP (mmol TE eq/g dw)
Breval Blanca	83.16 ± 6.93 ^b^	6.41 ± 0.66 ^b^	4.29 ± 0.53 ^ed^
Cuello Dama Blanca	20.84 ± 2.3 ^c^	3.73 ± 0.61 ^c^	4.14 ± 0.58 ^def^
El Quoti Lbied	14.28 ± 1.42 ^d^	3.59 ± 0.43 ^c^	1.09 ± 0.04 ^g^
Fassi	17.52 ± 0.98 ^cd^	5.96 ± 0.52 ^b^	6.06 ± 0.9 ^bc^
Ghoudan	17.78 ± 0.73 ^cd^	1.76 ± 0.48 ^d^	2.89 ± 0.33 ^ef^
Kadota	17.3 ± 1.27 ^cd^	2.43 ± 1.02 ^d^	2.55 ± 1.27 ^efg^
Nabout	88.1 ± 3.37 ^a^	4.44 ± 0.55 ^c^	10.65 ± 0.18 ^a^
Noukali	17.95 ± 1.47 ^cd^	8.04 ± 0.3 ^a^	7.44 ± 2.2 ^bc^
Palmeras	18.02 ± 1.96 ^cd^	1.43 ± 0.25 ^d^	4.95 ± 0.12 ^cd^
Snowden	14.76 ± 1.61 ^d^	1.75 ± 0.51 ^d^	3.53 ± 0.81 ^ef^
White Adriatic	15.21 ± 1.37 ^d^	2.11 ± 0.46 ^d^	1.97 ± 0.88 ^fg^
Mean	38.16 ± 2.12	2.78 ± 0.66	4.50 ± 0.71
ANOVA mean square	1967.25 ***	14.53 ***	18.51 ***

Average values ± standard errors of the mean are presented. Different letters (a–g) in columns represent statistically significant differences among cultivars at *p* < 0.05. *** denotes significant difference at *p* < 0.001.

**Table 6:** Matrix of coefficient correlations between all variables analyzed.

	Citric Acid	Malic Acid	SUCR	GLUC	FRUC	ABTS	DPPH	FRAP	TP	L*	c*	h*
Citric acid	1	0.488 **	0.518 **	0.388 *	0.441 *	0.141	0.548 **	0.125	−0.323	0.391 *	0.372 *	0.155
Malic acid		1	0.379 *	0.487 **	0.583 **	−0.226	0.115	0.146	−0.492 **	0.734 **	0.499 **	0.103
SUCR			1	−0.122	−0.052	0.031	−0.015	0.185	−0.178	0.240	0.255	0.295
GLUC				1	0.988 **	0.052	0.473 **	0.207	−0.015	0.496 **	0.350 *	−0.048
FRUC					1	0.078	0.455 **	0.221	−0.059	0.548 **	0.358 *	−0.021
ABTS						1	0.371 *	0.517 **	0.524 **	−0.185	−0.349	0.195
DPPH							1	0.473 **	−0.316	0.361 *	0.345	−0.168
FRAP								1	0.075	0.019	−0.193	0.050
TP									1	−0.558 **	−0.563 **	0.228
L*										1	0.855 **	−0.168
c*											1	−0.285
h*												1

*, ** denote significant differences at levels of 0.05 and 0.01 respectively. GLUC, glucose; FRUC, fructose; SUCR, sucrose; TP, total phenol.

**Table 7:** Eigenvectors of principal component axes from PCA analysis.

Variables	Components		
	1	2	3
Citric acid	**0.672**	0.236	0.403
Malic acid	**0.797**	−0.126	0.260
Sucrose	0.284		**0.856**
Glucose	**0.717**	0.359	−0.408
Fructose	**0.764**	0.355	−0.326
ABTS	−0.124	**0.853**	
DPPH	**0.587**	0.460	−0.189
FRAP	0.182	**0.694**	0.101
Total phenols	**−0.568**	0.548	−0.119
L*	**0.871**	−0.238	
c*	**0.760**	−0.430	
h*	−0.108	0.297	**0.626**
Percentage of variance	36.091	19.825	14.152
Cumulative percentage	36.091	55.916	70.068

Coefficients for which the absolute score value was under |0.1| were deleted; eigenvalues greater than |0.55| are marked in bold.

## Data Availability

The data presented in this study are available on request from the corresponding author.
